# Preclinical immunological evaluation of an intradermal heterologous vaccine against SARS-CoV-2 variants

**DOI:** 10.1080/22221751.2021.2021807

**Published:** 2022-01-07

**Authors:** Shengtao Fan, Kang Xiao, Dandan Li, Heng Zhao, Jingjing Zhang, Li Yu, Penglan Chang, Shuangli Zhu, Xingli Xu, Yun Liao, Tianjiao Ji, Guorun Jiang, Dongmei Yan, Fengyuan Zeng, Suqin Duan, Baicheng Xia, Lichun Wang, Fengmei Yang, Zhanlong He, Yang Song, Pingfang Cui, Xiaolei Li, Yaxing Zhang, Bangyi Zheng, Ying Zhang, Wenbo Xu, Qihan Li

**Affiliations:** a Institute of Medical Biology, Chinese Academy of Medicine Sciences & Peking Union Medical College, Yunnan Key Laboratory of Vaccine Research and Development for Severe Infectious Diseases, Kunming, 650118, China; b National Institute for Viral Disease Control and Prevention, China CDC, Beijing, 102206, China

**Keywords:** SARS-CoV-2, variants, intradermal administration, vaccine

## Abstract

The recent emergence of COVID-19 variants has necessitated the development of new vaccines that stimulate the formation of high levels of neutralizing antibodies against S antigen variants. A new strategy involves the intradermal administration of heterologous vaccines composed of one or two doses of inactivated vaccine and a booster dose with the mutated S1 protein (K-S). Such vaccines improve the immune efficacy by increasing the neutralizing antibody titers and promoting specific T cell responses against five variants of the RBD protein. A viral challenge test with the B.1.617.2 (Delta) variant confirmed that both administration schedules (i.e. “1 + 1” and “2 + 1”) ensured protection against this strain. These results suggest that the aforementioned strategy is effective for protecting against new variants and enhances the anamnestic immune response in the immunized population.

## Introduction

The COVID-19 pandemic caused by SARS-CoV-2, a member of the β-group of the coronavirus family, has infected approximately 180 million people and caused 3.8 million deaths since the end of 2019 [1]. The appearance of various SARS-CoV-2 variant strains worldwide has caused concern among the public [2–5]. There is an urgent need to develop a new generation of vaccines that enhance the protection against the new SARS-CoV-2 variants, stimulate the development of high levels of neutralizing antibodies, and can be manufactured rapidly to meet the demand created by new SARS-CoV-2 variants in a real-world situation with more than 1 billion people immunized worldwide by approximately 10 licensed vaccines [6].

Based on previously published data, several uncertainties exist. First, the best methods to induce and maintain a high level of neutralizing antibodies in immunized individuals are not clear. Previous animal studies have shown that a wide range of neutralizing antibody titers provide effective protection in viral challenge tests [7,8]. Surveys of convalescent serum from infected patients show a downward trend in neutralizing antibodies over six months [9,10]. Similarly, our previous clinical trial showed very low neutralizing antibody levels 6 months after immunization with two doses of an inactivated vaccine administered at an interval of 14 or 28 days [11]. Second, recent studies on isolated variants have suggested that higher titers are required in the immune sera to enable neutralization of variant strains compared to prototype strains [12–15]. Based on the aforementioned data, a vaccine that has the capacity to maintain neutralizing antibody levels for a prolonged duration of time and stimulate enhanced specific immune recognition of variants will be a good candidate for further evaluation. This is because of the challenges of rapid appearance of variants and substantial time required to develop vaccines and obtain approval for and conduct three phases of clinical trials for new vaccines.

In the present study, a new heterologous vaccine strategy was evaluated, which involved the intradermal administration of 1/5–1/3 of the quantity of antigen authorized for use in clinical trials of inactivated vaccines, followed by a booster dose of eukaryotically-expressed S1 protein (K-S) with 6 mutated amino acids, similar to the sequences in B.1.351, B.1.617, and B.1.1.7 strains [16,17]. The inactivated vaccine was being evaluated in a phase III trial and approved for emergency use in China. Two doses of the vaccine (150 U/dose) were administered at an interval of 14 or 28 days associated with Aluminum (Al) [18]. The results suggest that administering a booster dose of the inactivated vaccine against the optimized S1 protein can elicit a high level of neutralizing antibodies and a specific T cell response with the ability to cross-neutralize three variant antigens. Importantly, a viral challenge test with the B.1.617.2 strain showed that hACE2 transgenic mice immunized with this heterologous vaccine were capable of preventing and restraining viral infection and proliferation in tissues; therefore, no pathological lesions were observed in these mice. These observations suggest that the intradermal heterologous vaccine can not only ameliorate the immune effect, probably by improving the innate immunity and activating the adaptive immunity, but also allow the emergency development of vaccines using a cell seed library containing strains transfected with eukaryotic vectors expressing S1 protein, including various variants preconstructed based upon genetic prediction of sequences with a high frequency of mutations.

## Materials and methods

### Cells and virus

The Vero cell strain WHO Vero 10-87 used to produce the SARS-CoV-2 inactivated vaccine and detect neutralizing antibodies was provided by the WHO [18,19]. The cells were grown in MEM-5% FCS medium in culture plates to form monolayers for various assays or in microcarriers in bioreactors using an MEM-based protocol to produce inactivated vaccines. CHO cells expressing the S1 protein with mutated sites (Suqiao Co., Suzhou, China) were grown in RPMI 1640–8% FCS medium using a bioreactor-based standard protocol [20]. Two SARS-CoV-2 strains with S protein sequences from the prototype strains (Wuhan strain) KMS-1 (MT226610.1) and KMS-2 were isolated from the Yunnan Infectious Hospital in January 2020. The variant strains B.1.351, B.1.617.2 (Delta), and Wuhan used for cross-neutralization assays and challenge tests were provided by the National Institute for Viral Disease Control and Prevention, China.

### SARS-CoV-2 inactivated vaccine

The SARS-CoV-2 inactivated vaccine was developed by the Institute of Medical Biology (IMB), Chinese Academy of Medical Sciences (CAMS). In brief, the virus strain (KMS-1) was inoculated into a medium containing Vero cells. Dual inactivation of the virus harvested from Vero cells was performed with formaldehyde (HCHO, 1:4000; 48 h) to partially disrupt the viral membrane, followed by beta-propiolactone (BPL, 1:2000; 48 h) to disrupt the structure of the viral genome. The viral antigen content was measured using enzyme-linked immunosorbent assay (ELISA). The vaccine contained 150 U of inactivated SARS-CoV-2 viral antigen adsorbed to the adjuvant Al(OH)_3_ (0.0875 mg of Al), suspended in 0.5 ml of buffered saline and administered intramuscularly to individuals aged 18–80 years.

### Recombinant antigen (K-S) from the S1 protein with six mutated sites

The optimized S1 (K-S) sequence with six mutated sites (G1251 T [K417N], T1355G [L452R], G1450C [E484Q], A1501 T [N501Y], A1841G [D614G], and C2042G [P681R]) was designed for construction of a eukaryotic expression vector (patent application submitted). The plasmid was transfected into CHO cells according to the standard protocol. The identified and screened clone was grown in RPMI 1640 medium for the construction of libraries of primary, main, and working seeds, followed by qualification assays based on the requirements of the Chinese FDA. The K-S protein was expressed under optimized conditions in middle-scale production. After purification by chromatography, the purified antigen (10 µg/dose) was formulated with Al(OH)_3_ adjuvant (0.0175 mg of Al/dose) for the immunological study.

### Preparation of the immune serum against K-S protein

Rabbits were intramuscularly administered three doses of K-S antigen attached to Al(OH)_3_ adjuvant at intervals of 14 days. Immune serum was obtained on day 14 after the final injection.

### Western blots

Proteins were separated by 12% SDS–PAGE and transferred to polyvinylidene difluoride (PVDF) membranes. The membranes were blocked with 5% BSA-TBST (Sigma–Aldrich, St. Louis, MO, USA). Then, the membranes were treated with convalescent or immunized serum and HRP-conjugated goat anti-human IgG (H + L) (4A Biotech, Beijing, China) or HRP-conjugated goat anti-rabbit IgG (Sigma, Shanghai, China). Finally, the PVDF membrane was covered with ECL ultrasensitive chemiluminescence reagent (Beyotime, Jiangsu, China) and placed in a Bio–Rad gel imager for exposure and colour development. The recombinant S1 protein (Sanyou Biopharmaceuticals Co., Ltd., Shanghai, China), the unmutated spike RBD protein (SinoBiological Co., Ltd, Beijing, China), and the single-point mutation RBD protein (K417N, L452R, E484Q, N501Y, N439 K, and A520 V; SinoBiological Co., Ltd) were used in this study.

### Electron microscopy

Purified inactivated SARS-CoV-2 preparations were co-incubated with convalescent serum or monoclonal antibody (mAb) against the S protein (mAb-S) or N protein (mAb-N) (Solarbio, Beijing, China) at 37°C for 24 h, stained with 1% phosphotungstic acid, and observed using a transmission electron microscope (Hitachi, Kyoto, Japan).

### Animal experiments and ethical approval

The animal experiment was performed according to the principles in the “Guide for the Care and Use of Laboratory Animals” and “Guidance for Experimental Animal Welfare and Ethical Treatment”. The protocols were approved by the Experimental Animal Management Association of the IMB, CAMS (no.: DWSP 202004 014-1/2). All animals were cared for by veterinarians at the IMB, CAMS.

### Local innate immune response in injected tissues

In total, 48 C57 female mice (Shanghai Model Organisms Center, Inc., Shanghai, China) were divided into 4 subgroups with 12 mice each. Mice in the ID-V group received a single intradermal injection of 30 U of inactivated vaccine. Mice in the ID-K group received a single intradermal injection of 10 μg of K-S protein. Mice in the IM-V group received a single intramuscular injection of 30 U of inactivated vaccine. Mice in the IM-K group received a single intramuscular injection of 10 μg of K-S protein. At 12, 24, and 48 h after immunization, the immunized mice in the ID-V and IM-V groups were euthanized, and local skin and muscle tissues were obtained (Fig. S1a). Blood samples from the remaining mice were obtained on day 14 after booster immunization.

### Comparison of intradermal and intramuscular injections

Eight macaques aged 2–3 years were divided into two subgroups with four macaques in each subgroup. In the ID group, macaques received a single intradermal injection of 30 U of inactivated vaccine and blood was obtained for antibody assays on day 14 after immunization. In the IM group, macaques received a single intramuscular injection of 150 U of inactivated vaccine at an interval of 14 days and blood was obtained for antibody assays on day 14 after booster immunization (Fig. S1b).

### Animal immune experiment

**Figure 1:**
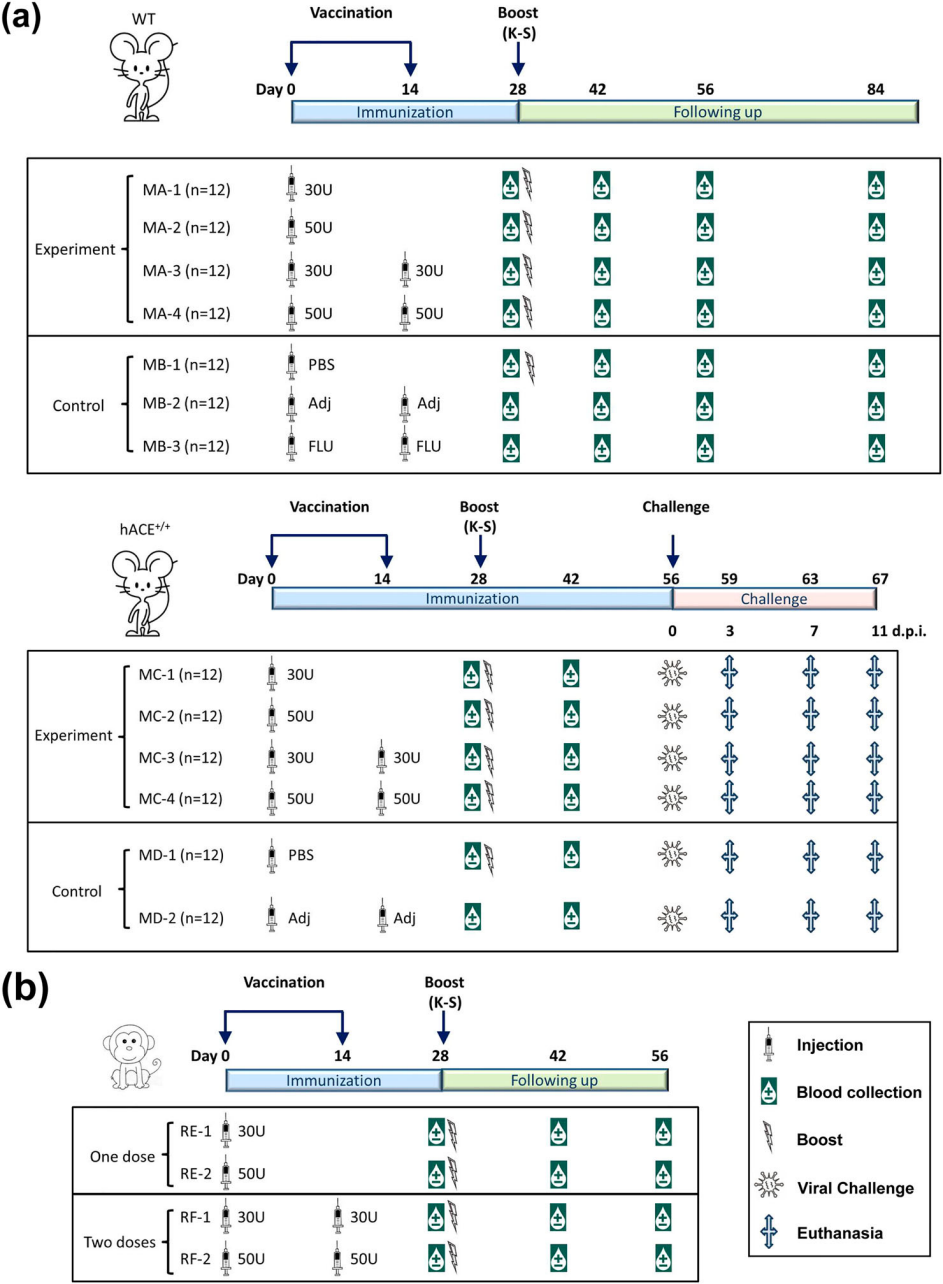
. Design of animal immunization schedule and viral challenge. (a) Mouse immunity and challenge experiment. This experiment included hACE2 transgenic mice (MC and MD groups) and WT mice (MA and MB groups). The mice received intradermal injections (ID; syringe signs) with different doses (30 or 50 U) of inactivated SARS-CoV-2 vaccine, PBS, adjuvant (Adj), and inactivated influenza vaccine (FLU) through different procedures (one or two injections at an interval of 14 days). Some mice were boosted (thunder-like signs) with the K-S antigen (10 μg/dose). Blood samples (water drop-like signs) were obtained on days 28 and 42 after the first immunization in hACE2 transgenic mice (MCs and MBs), and on days 28, 42, 56, and 84 after the first immunization in WT mice (MAs and MBs) for antibody assays. Viral challenge (virus-like signs) was performed on day 56 after the first immunization in hACE2 transgenic mice (MAs and MBs). At days 3, 7, and 11 after the viral challenge, three mice from each group were euthanized (cross signs) for viral load measurement and pathological observation. MBs were the control groups for MAs, and MDs were the control groups for MCs (n = 12 per subgroup). (b) Rhesus macaque immunity experiment. The macaques received intradermal injections (ID; syringe signs) of different doses (30 or 50 U) of inactivated SARS-CoV-2 vaccine through different procedures (one or two injections at an interval of 14 days). The macaques were boosted (thunder-like signs) with the K-S antigen (10 μg/dose) on day 28 after the first immunization. Blood samples (water drop-like signs) were obtained on days 28, 42, and 56 after the first immunization for antibody assays (n = 4 per subgroup).

Mouse immunogenicity study:

- Group A (MA) mice, which included 48 C57 female mice (wild-type, WT), were divided into 4 subgroups with 12 mice in each subgroup. In MA-1, the mice received a single intradermal injection of 30 U of inactivated vaccine, followed by a booster dose with the expressed K-S antigen (10 μg/dose) at day 28 after the first injection. In MA-2, the mice received a single intradermal injection of 50 U of inactivated vaccine per mouse, followed by the same procedure described for MA-1. In MA-3, the mice the mice received two intradermal injections of 30 U of inactivated vaccine per mouse at an interval of 14 days and booster dose with the K-S antigen (10 μg/dose) at day 28 after the first injection (i.e. day 14 after the second dose of the inactivated vaccine). In MA-4, the mice received two intradermal injections of 50 U of inactivated vaccine per mouse at an interval of 14 days and booster with the K-S antigen (10 μg/dose) at day 28 after the first injection (i.e. day 14 after the second dose of the inactivated vaccine). Blood samples were obtained on days 0 (28 days after the first injection), 14 (42 days after the first injection), 28 (56 days after the first injection), and 56 (84 days after the first injection) after booster immunization for antibody assays (Figure 1a).
- Group B (MB) mice, which included 36 C57 female mice (WT), were divided into 3 subgroups with 12 mice in each subgroup. In MB-1, the mice received an intradermal injection of PBS (10 mmol) followed by K-S antigen (10 μg/dose) on day 28 after the first injection. In MB-2, the mice received two intradermal injections of Al(OH)_3_ adjuvant (0.0175 mg of Al/dose) at an interval of 14 days. In MB-3, the mice received two intradermal injections of the inactivated influenza vaccine (Hualan Bio, Xinxiang, China) at an interval of 14 days. Blood samples were obtained on days 0 (28 days after the first injection), 14 (42 days after the first injection), 28 (56 days after the first injection), and 56 (84 days after the first injection) after booster immunization for antibody assays (Figure 1a).
- Group C (MC) mice, which included 48 hACE2 transgenic female mice (hACE2^+/+^; Shanghai Model Organisms Center, Inc.), were divided into 4 subgroups with 12 mice in each subgroup. In MC-1, the mice received a single intradermal injection of 30 U of inactivated vaccine per mouse (antigen quantity was equal to 1/5 of that used for intramuscular injection) and booster with the expressed K-S antigen (10 μg/dose) at day 28 after the first injection. In MC-2, the mice received a single intradermal injection of 50 U of inactivated vaccine per mouse (antigen quantity equal to 1/3 of that used for intramuscular injection) followed by the same methods described for MC-1. In MC-3, the mice received two intradermal injections of 30 U/dose of the inactivated vaccine at an interval of 14 days, and booster with K-S antigen (10 μg/dose) at day 28 after the first injection (i.e. day 14 after the second dose of the inactivated vaccine). In MC-4, the mice received two intradermal injections of 50 U/dose at an interval of 14 days and booster with the K-S antigen (10 μg/dose) at day 28 after the first injection (day 14 after the second dose of the inactivated vaccine). Blood samples were obtained on days 0 (28 days after the first injection) and 14 (42 days after the first injection) after booster immunization for antibody assays (Figure 1a).
- Group D (MD) mice, which included 24 hACE2 transgenic female mice (hACE2^+/+^), were divided into 2 subgroups with 12 mice in each subgroup. In MD-1, the mice received intradermal injection of PBS (10 mmol) followed by K-S antigen (10 μg/dose) on day 28. In MD-2, the mice received two intradermal injections of Al(OH)_3_ adjuvant (0.014–0.021 mg Al/dose) at an interval of 14 days. Blood samples were obtained on days 0 (28 days after the first injection) and 14 (42 days after the first injection) after booster immunization for antibody assays (Figure 1a).
- Observation set: For control observation, an additional 10 mice from the MC and MD groups were immunized according to the immunization programme described above.

Rhesus macaque study:

— Group E (RE), which included eight macaques aged 2–3 years, was divided into two subgroups with four macaques in each subgroup. In RE-1, macaques received a single intradermal injection of 30 U of inactivated vaccine and booster with the K-S antigen (10 μg/macaque). In RE-2, macaques received a single intradermal injection of 50 U of inactivated vaccine and booster with the K-S antigen (10 μg/macaque).
— Group F (RF), which included eight macaques, was divided into two subgroups with four macaques in each subgroup. In RF-1, macaques received two intradermal injections of 30 U of inactivated vaccine at an interval of 14 days and booster with the K-S antigen (10 μg/macaque). In RF-2, macaques received two intradermal injections of 50 U of inactivated vaccine per macaque at an interval of 14 days and booster with the K-S antigen (10 μg/macaque) on day 14 after the second dose of the inactivated vaccine (Figure 1b).
— In addition, three untreated healthy macaques were included in the control group.

### Transcriptional profile of innate immune signaling molecules in local tissues inoculated with the vaccine

Skin tissue samples were homogenized with a Tissue Lyser II system (Qiagen, Hilden, Germany), and total RNA was extracted from these tissue samples using TRIzol-A^+^ Reagent (Tiangen, Beijing, China) according to the manufacturer’s protocol. The One Step TB Green Prime Script PLUS RT–PCR Kit (TaKaRa, Shiga, Japan) was used for q-RTPCR quantification on the BIO-RAD iCycler Thermal Cycler. The detection primers used for mRNA profiling are shown in Table S1.

### Fluorescence confocal microscopy

Local skin tissues from immunized mice were immediately frozen in liquid nitrogen. The tissue sections were embedded, sliced, fixed, and blocked using 5% bovine serum albumin (BSA). For detection of the viral antigen, the sections were sequentially incubated with a primary mouse anti-SARS-CoV-2 spike antibody (SinoBiological Co., Ltd) and an AlexaFluor 647-conjugated goat anti-mouse IgG secondary antibody (Invitrogen, Carlsbad, CA, USA). Dendritic cells were detected using anti-CD11c antibody (Abcam, Cambridge, UK) and Alexa Fluor® 488 goat anti-rabbit IgG secondary antibody (Invitrogen). The cell nuclei were detected using DAPI. Fluorescence was visualized and analyzed using a confocal microscope (TCS SP2, Leica).

### Neutralization assay for viral strains

Heat-inactivated serum was serially diluted and co-incubated with live virus (100 lgCCID_50_/well) for 2 h at 37°C. Then, 100 μl of the Vero cell suspension (10^5^ cells/ml) was added to the mixture, followed by incubation at 37°C in a 5% CO_2_ atmosphere for 7 days. Cytopathic effects were assessed using an inverted microscope (Nikon) to determine the serum neutralizing antibody titer. The geometric mean titers (GMTs) of the neutralizing antibodies were measured.

### ELISA

ELISA was conducted with antibodies against the S and N proteins. S and N proteins (Sanyou Biopharmaceuticals Co.) were used to coat the ELISA plates (Corning Costar; Corning, NY, USA) at a concentration of 5 μg/well, and the plates were incubated overnight at 4°C. The plates were blocked with 5% BSA, incubated with serum samples, and incubated with an HRP-conjugated antibody (Abcam). The immune complexes were visualized using TMB substrate (Solarbio) as described previously [21]. The absorbance at 450 nm was measured using an ELISA plate reader (Gene Company, Beijing, China). The antibody serum samples that yielded OD values at least 2.1-fold higher than that of the negative control at a test sample dilution of 1:400 were considered positive. The endpoint titer (ET) was defined as the highest serum dilution that yielded a positive OD value. The GMT was calculated as the geometric mean of the ETs of the positive serum samples in each group.

### Elispot assay

Peripheral blood mononuclear cells were isolated from blood using a lymphocyte isolation technique (Ficoll-Paque Premium; GE Healthcare, Piscataway, NJ, USA). An ELISPOT assay was performed with a Mouse or Monkey IFN-γ ELISPOT Kit (Mabtech, Cincinnati, OH, USA), according to the manufacturer’s protocol. The samples were plated in duplicate wells. Different stimuli, namely, four single-point mutation RBD proteins (K417N, L452R, E484Q, and N501Y; SinoBiological Co., Ltd), a two-point mutation RBD protein (L452R + E484Q; SinoBiological Co., Ltd), and recombinant N protein (Sanyou Biopharmaceuticals Co., Ltd.), were added to separate wells. The positive control was phytohemagglutinin (PHA). The plate was incubated at 37°C for 24 h. Then, the cells were removed and the spots were developed. The coloured spots were counted using an ELISPOT reader (CTL, Shaker Heights, OH, USA).

### Viral challenge test with the B.1.617.2 strain in immunized hACE2 transgenic mice

In the MC, MD, and observation groups, immunized hACE2 transgenic mice were infected with the B.1.617.2 strain (1 × 10^3^ CCID_50_/dose) via a nasal spray under ABSL-3 laboratory conditions on day 28 after booster immunization with the K-S antigen (day 56 after the first immunization). The animals were evaluated daily for clinical signs. Pharyngeal and nasal secretion samples were obtained daily after infection. On days 3, 7, and 11 after the viral challenge, three mice from each group were euthanized for viral load measurement and pathological observation. The remaining mice were euthanized after completion of the experiment.

### Histopathological detection of mouse tissues in the challenge test

The organs of experimental animals were fixed with 10% formalin, embedded in paraffin, sliced into 4-μm sections, and stained with hematoxylin and eosin. The morphology was assessed using an inverted microscope (Nikon).

### Viral load measurement by q-RTPCR

Total RNA was extracted from blood and tissue samples using TRIzol reagent (Tiangen). The q-RT–PCR was performed using a Novel Coronavirus (SARS-CoV-2) Nucleic Acid Isothermal Amplification Rapid Detection Kit (Chinese Center for Disease Control and Prevention) according to the standard protocol. The primers used for q-RT–PCR were selected to amplify the N and ORF1ab sequences in the SARS-CoV-2 genome. The viral copy numbers of the samples were quantified according to the standard protocol (National Institute of Metrology, China).

### Statistical analysis

Data are shown as mean or geometric mean and standard deviation (SD). The differences among the groups were evaluated by two-way analysis of variance (ANOVA). The survival data were analyzed using the log-rank test. GraphPad Prism software (San Diego, CA, USA) was used for statistical analyses. P < 0.05 was considered statistically significant.

## Results

### Antigenic identification of the recombinant K-S protein with six mutated sites and inactivated vaccine

**Figure 2:**
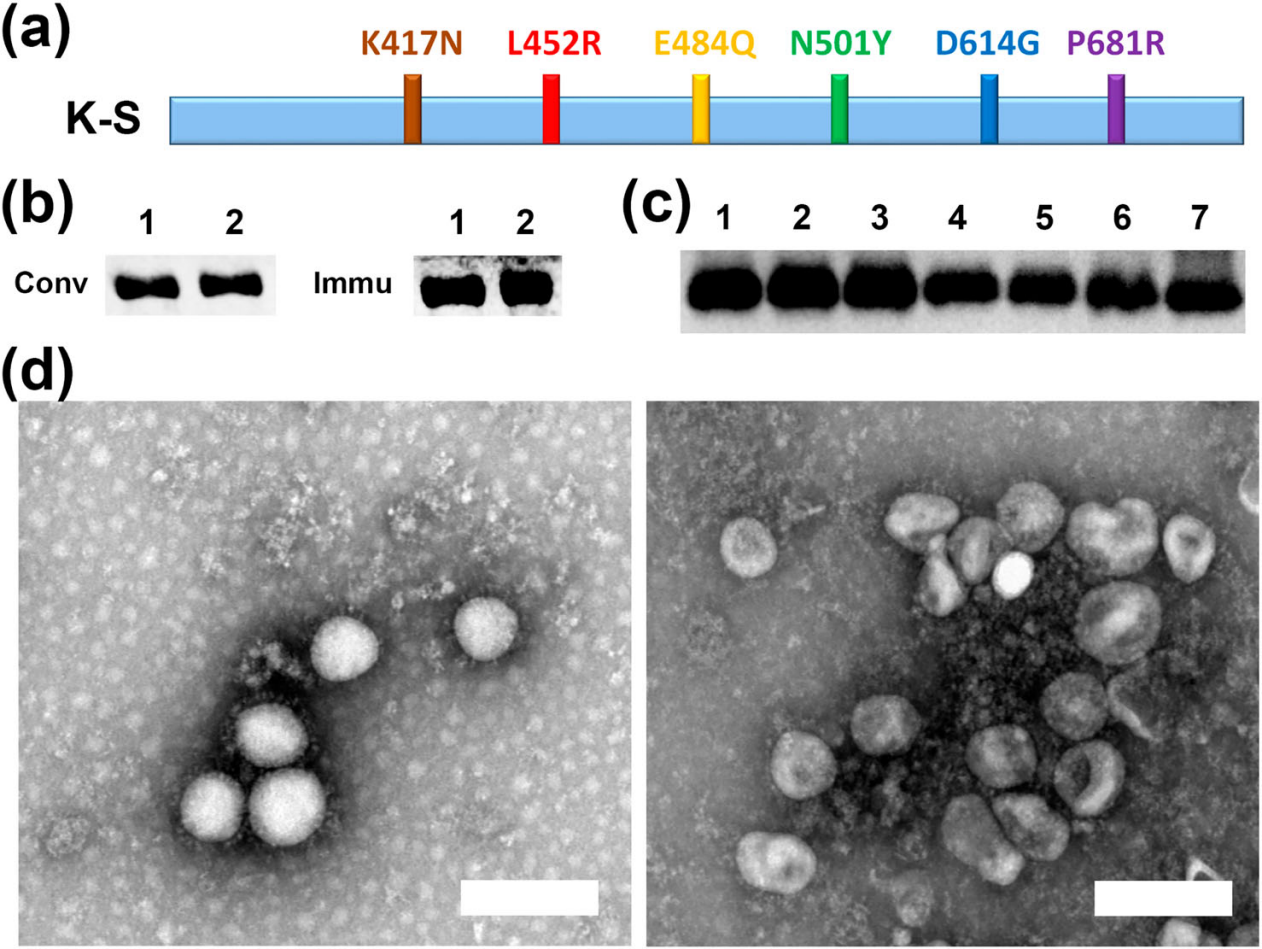
. Immunological characterization of the recombinant K-S S1 protein and inactivated vaccine. (a) An optimized sequence of the S1 protein (K-S) with six mutated sites (N501Y, K417N, E484Q, L452R, P681R, and D614G). (b) K-S protein was recognized by both convalescent serum (Conv) from confirmed COVID-19 patients and immune serum (Immu) from vaccinated individuals in a clinical trial. Lane 1, S1 recombinant protein; lane 2, K-S protein. Proteins separated by SDS–PAGE and transferred to PVDF membranes were identified using western blotting. (c) Serum from rabbits immunized with the K-S protein recognized the single-point mutation RBD protein (lanes 1–6) and the unmutated RBD protein (lane 7). Lanes: 1, K417N; 2, L452R; 3, E484Q; 4, N501Y; 5, N439 K; 6, A520 V; 7, Spike RBD protein. Mutated or unmutated proteins were recognized by K-S-immunized serum through western blotting. (d) Electron micrograph of S/N particles. Exposure of antibody recognition epitopes of SARS-CoV-2 inactivated by HCHO-BPL. The viral antigens inactivated by HCHO-BPL were co-incubated with the anti-S (left) or anti-N (right) antibody for 2 h at 37°C in PBS buffer and observed using an electron microscope (25,000×). Bar, 200 nm.

Based on recent data on variant strains, an optimized sequence of the S1 protein with six mutated sites (N501Y, K417N, E484Q, L452R, P681R, and D614G) found in B.1.351, B.1.617.2, and B.1.1.7 variants was named K-S (Figure 2a) [12,22–24]. The specific antigenicity of the recombinant protein was first investigated by immunoblotting with convalescent serum from confirmed COVID-19 patients in Wuhan and immune serum from vaccinated individuals in our clinical trial of the inactivated vaccine [18]. The results showed that this protein was recognized by both sera (Figure 2b), while the serum collected from rabbits immunized with three injections of protein attached to Al(OH)_3_ adjuvant at intervals of 14 days was capable of interacting with S protein with mutated sites (Figure 2c). These data confirmed the specific antigenicity of the K-S protein. The inactivated vaccine used in this study was developed by our laboratory and its protective efficacy is being evaluated in a phase III clinical trial; this vaccine was produced through two technical steps of inactivation with formaldehyde and β-propiolactone to expose the viral S and N proteins. The S and N antigenicity of this inactivated vaccine was confirmed by immune-electron microscopy and phase I and II clinical trials (Figure 2d) [18,19].

### Activation of the innate immune response in epithelial tissues inoculated with K-S protein or intradermal inactivated vaccine

**Figure 3:**
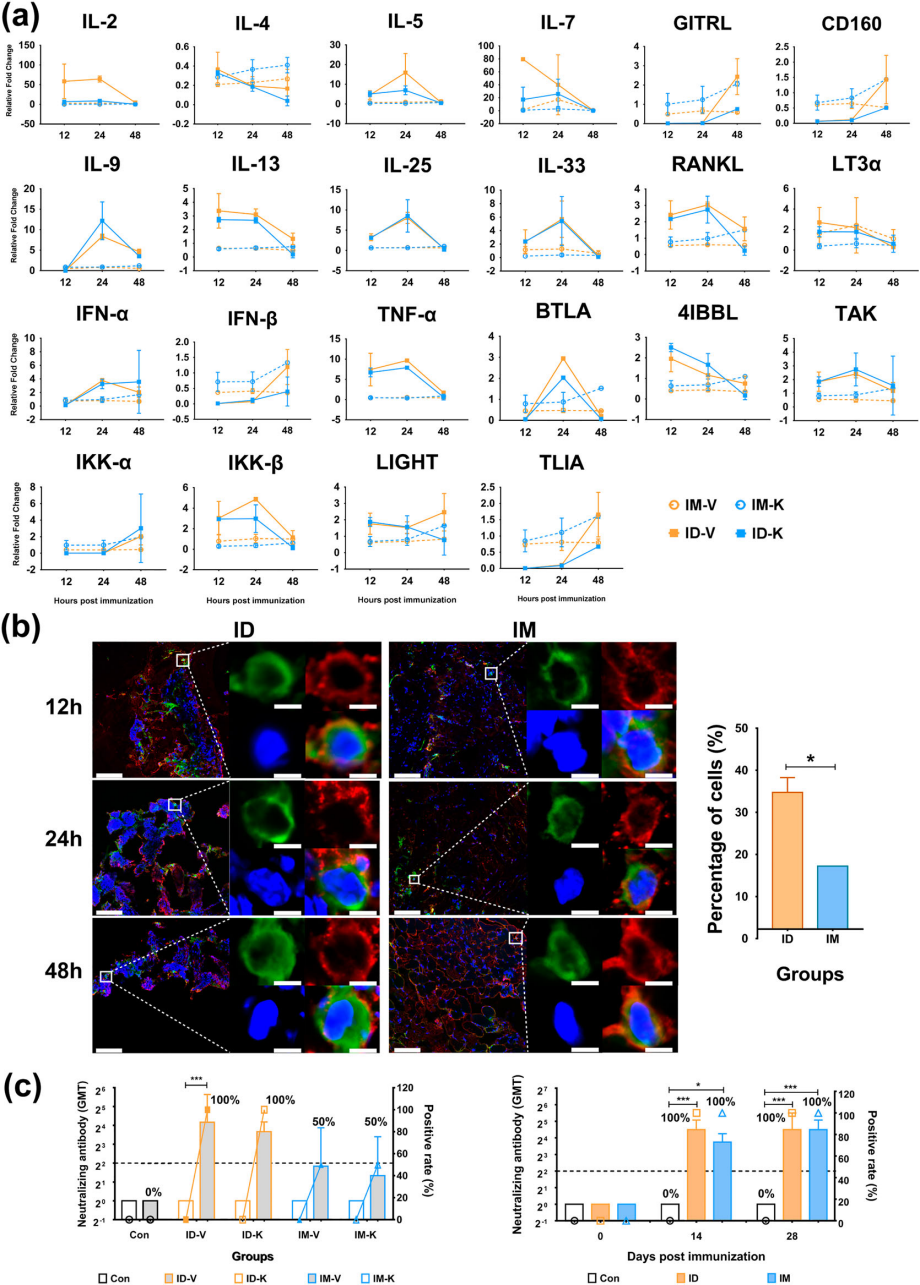
. More powerful immunity was induced by viral antigens after ID immunization than IM immunization. (a) Dynamic alteration of the mRNA profile related to immune regulatory signaling molecules in the local skin tissue elicited by the SARS-CoV-2 inactivated vaccine or the K-S protein via ID or IM immunization in WT mice. The RNA expression levels were used as an internal control for normalization of housekeeping GAPDH mRNA expression using the comparative Ct method (ΔΔCt). (b) The co-localization of viral antigen and dendritic cells in skin tissue elicited by the SARS-CoV-2 inactivated vaccine or K-S protein via ID (ID-V) or IM (IM-V) groups at 12, 24, and 48 hr after immunization in WT mice. Nuclear staining (DAPI), viral antigen (red), and dendritic cells (green) are shown. Bar, 6 μm. The bar graph (right) shows the statistical analysis of co-localization with viral antigen and DCs at 24 hr after immunization. The percentage of co-localized cells in the total DCs was based on the observation of 60 fields. (c) Neutralizing antibodies induced by the SARS-CoV-2 inactivated vaccine or K-S protein via ID or IM immunization in mice (left) and monkeys (right). The live viral strain is KMS-2 (Wuhan strain). The antibody titer is shown as columns, and the positive rate (%) is shown as a line and marked in the bar graph. In mice (left), the unfilled column shows the titer before immunization (at day 0), and the gray filled column shows the titer at 14 days after the first dose of immunization. Three samples from each subgroup were obtained at each time point. Statistical significance was assessed by two-way ANOVA. *, p < 0.05, ***, p < 0.001 versus before immunization (in mice, n = 12 per subgroup) or the control group (in monkeys, n = 4 per subgroup).

Data from a previous immunological study of viral vaccines suggested that the intradermal vaccination strategy could not only reduce the quantity of antigen required to induce immunity [25], but also enhance antiviral immunity through activation of the innate immune response followed by the adaptive immune response [26,27]. In the current study, a licensed microneedle apparatus for intradermal injection enabled the injection of vaccine antigens into epithelial tissues at a depth of 0.6 mm [28]. Our observations suggest that the local skin tissue of mice inoculated with SARS-CoV-2 inactivated vaccines or K-S proteins demonstrated more active dynamic alterations in the mRNA profile related to immune regulatory signaling molecules compared to local muscle tissue. These immune regulatory signaling molecules include IFN-α, TNF-α, RANKL, BTLA, LIGHT, IKKβ, 4IBBL, and interleukins such as IL-5, IL-9, IL-13, IL-25, and IL-33 (Figure 3a). Further observations using fluorescence confocal microscopy suggested that the co-localization rate of antigens from the inactivated vaccine or K-S protein and dendritic cells in skin tissue was 2–3-fold higher than that in muscle tissue after the injection (Figure 3b). These data suggest that the efficacy of antigens in activating the innate immune system in epithelial tissue was higher than that in muscle tissue; therefore, the adaptive immunity induced by the innate response was enhanced to elicit a stronger antibody response after intradermal immunization. Our neutralizing antibody assay indicated that the mice with intradermal immunization with a single dose of inactivated vaccine (30 U/dose) or K-S antigen (10 μg/dose) showed 100% seroconversion with GMTs of 20–30 compared to 50% seroconversion with GMTs of 3­–4 in mice intramuscularly immunized with a single dose of inactivated vaccine or K-S antigen (Figure 3c). These data confirmed the usefulness of intradermal immunization with the SARS-CoV-2 vaccine antigen.

### Antibody response elicited by sequential intradermal immunization with the inactivated vaccine and K-S antigen

**Figure 4:**
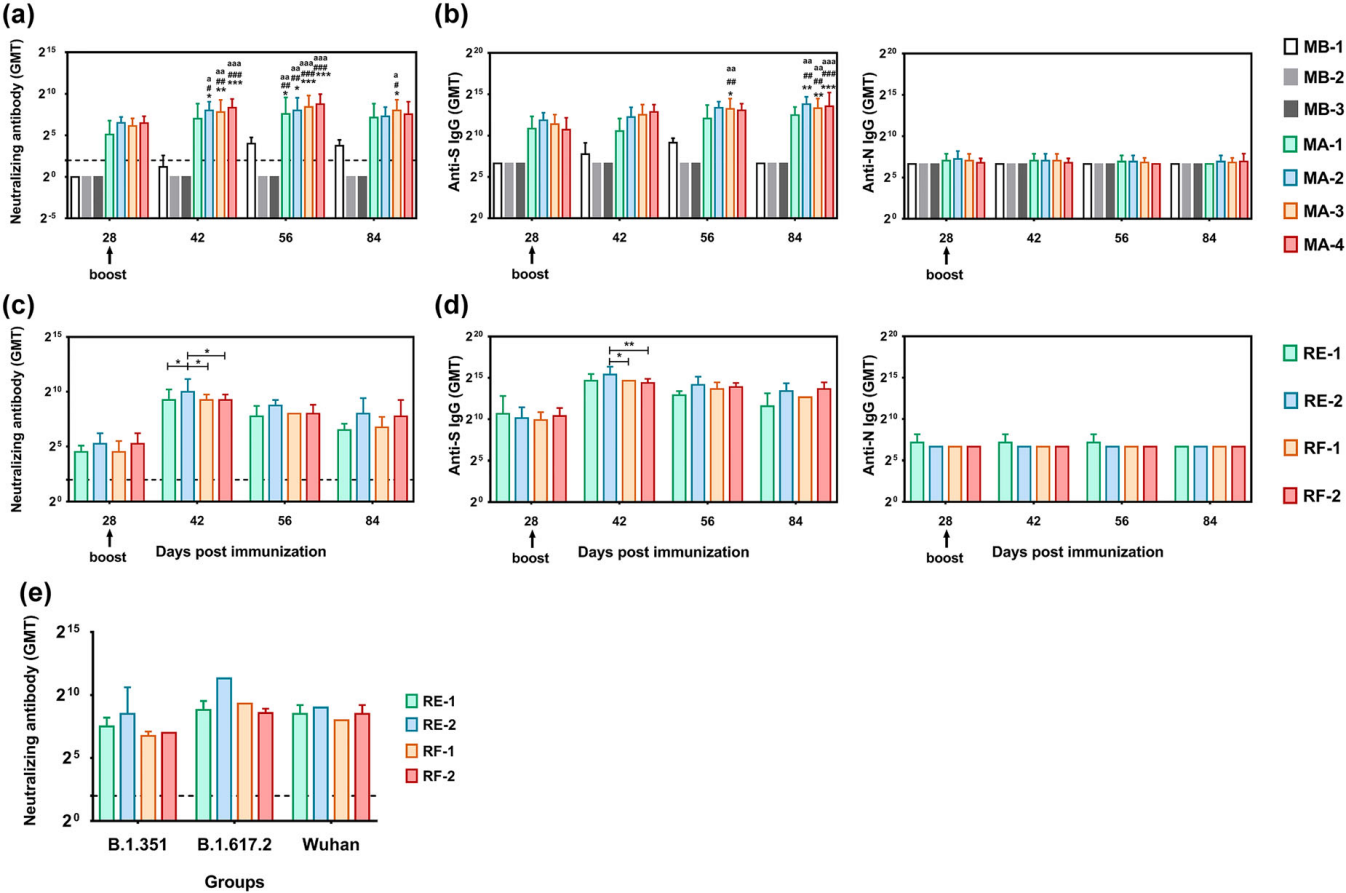
. Heterologous vaccine strategy using ID immunization enables the induction of high levels of NAb against various variant strains. (a) Neutralizing antibodies recalled by boosting with the K-S protein in mice immunized with the inactivated vaccine. The live viral strain is KMS-2 (Wuhan strain). (b) The levels of anti-S and anti-N antibodies after boosting with the K-S protein in mice immunized with the inactivated vaccine using ELISA. (c) The level of neutralizing antibodies induced by the inactivated vaccine and K-S protein in macaques. The live viral strain is KMS-2 (Wuhan strain). (d) The levels of anti-S and anti-N antibodies induced by the inactivated vaccine and K-S protein in macaques using ELISA. (e) Cross-neutralization ability of the *in vitro* antibodies induced by the inactivated vaccine and K-S protein in macaques. Blood samples were obtained on day 84 after the first immunization. The three live strains were B.1.351, B.1.617.2, and Wuhan. Statistical significance was assessed by two-way ANOVA. *, p < 0.05; **, p < 0.01;***; p < 0.001 versus MB-1 group; #, p < 0.05; ##, p < 0.01; ###, p < 0.001 versus MB-2 group; a, p < 0.05; aa, p < 0.01; aaa, p < 0.001 versus MB-3 group in WT mice (a-b; n = 12 per subgroup). *, p < 0.05; **, p < 0.01 versus the control group in monkeys (c-e; n = 4 per subgroup).

Based on the immunological observation of the SARS-CoV-2 inactivated vaccine and K-S antigen administered by intradermal immunization in animals and recently reported information about variant strains, our work aimed to increase the levels of neutralizing antibodies associated with specific recognition of variant antigens. We designed an intradermal immunization strategy involving one or two doses of inactivated vaccine at a dose of 30 or 50 U/dose and booster with the K-S protein (10 μg). This heterologous vaccine was evaluated in C57 mice, hACE2 transgenic mice, and rhesus macaques (Figure 1a, b). First, neutralizing antibody assays showed that the intradermal booster with the S1 protein with mutated sites stimulated the production of higher levels of neutralizing antibodies compared to the inactivated vaccine in immunized mice, regardless of the antigen dose or number of vaccinations (Figure 4a). In C57 mice, the neutralizing antibody levels after two doses of inactivated vaccine (50 U/dose) showed an upward trend from day 0 (28 days after the first immunization) to 14 (42 days after the first immunization) after the booster immunization with K-S compared to mice immunized with 30 U/dose, with a GMT of 88.44 to 322.54 and 70.20 to 222.86, similar to the trend in mice with a single intradermal injection of 30 or 50 U of the inactivated vaccine (Figure 4a). ELISA antibody detection in these mice showed a similar trend (Figure 4b). Similar trends were observed in hACE2 transgenic mice subjected to the viral challenge test (Fig. S2). Furthermore, the neutralizing antibody level was maintained until day 28 (56 days after the first immunization) and showed a slight decrease on day 56 (84 days after the first immunization) post-K-S antigen booster after reaching GMTs of 430.54–186.81 in C57 mice in the MA-4 group (Figure 4a). ELISA showed that the antibody levels exhibited similar alterations during the observation period, in which the GMTs of the S antibody reached 8542.97–11942.8 in the MA-4 group (Figure 4b). The neutralizing antibody assay in immunized macaques also indicated that a single intradermal immunization of 30 or 50 U of inactivated vaccine and a booster of K-S antigen was capable of eliciting neutralizing antibodies, with GMTs of 608.87 and 1024 in subgroups RE-1 and RE-2, respectively, on day 14 after the booster immunization (42 days after the first immunization); the upward trend was maintained for 1.5 months (Figure 4c). Interestingly, two intradermal immunizations with 30 or 50 U of inactivated vaccine and a booster of K-S antigen in subgroups RF-1 and RF-2 led to GMTs of neutralizing antibodies, with the same GMTs of 608.87 in the two subgroups; these titers were maintained until day 56 (84 days after the first immunization) after the booster immunization (Figure 4c). ELISA showed that the antibody levels exhibited similar alterations during the observation period, with GMT peaks of 25600 and 21526.9 in the RF-1 and RF-2 groups, respectively (Figure 4d). Importantly, a cross-neutralization assay showed that the immune sera induced in subgroups RE and RF enabled neutralization of the three strains, Wuhan, B.1.351, and B.1.617.2, with similar titers (Figure 4e). These data confirmed the immunological efficacy of our heterologous vaccine strategy.

### Specific T cell responses against variant antigens were elicited in animals immunized with the heterologous vaccine strategy

**Figure 5.**
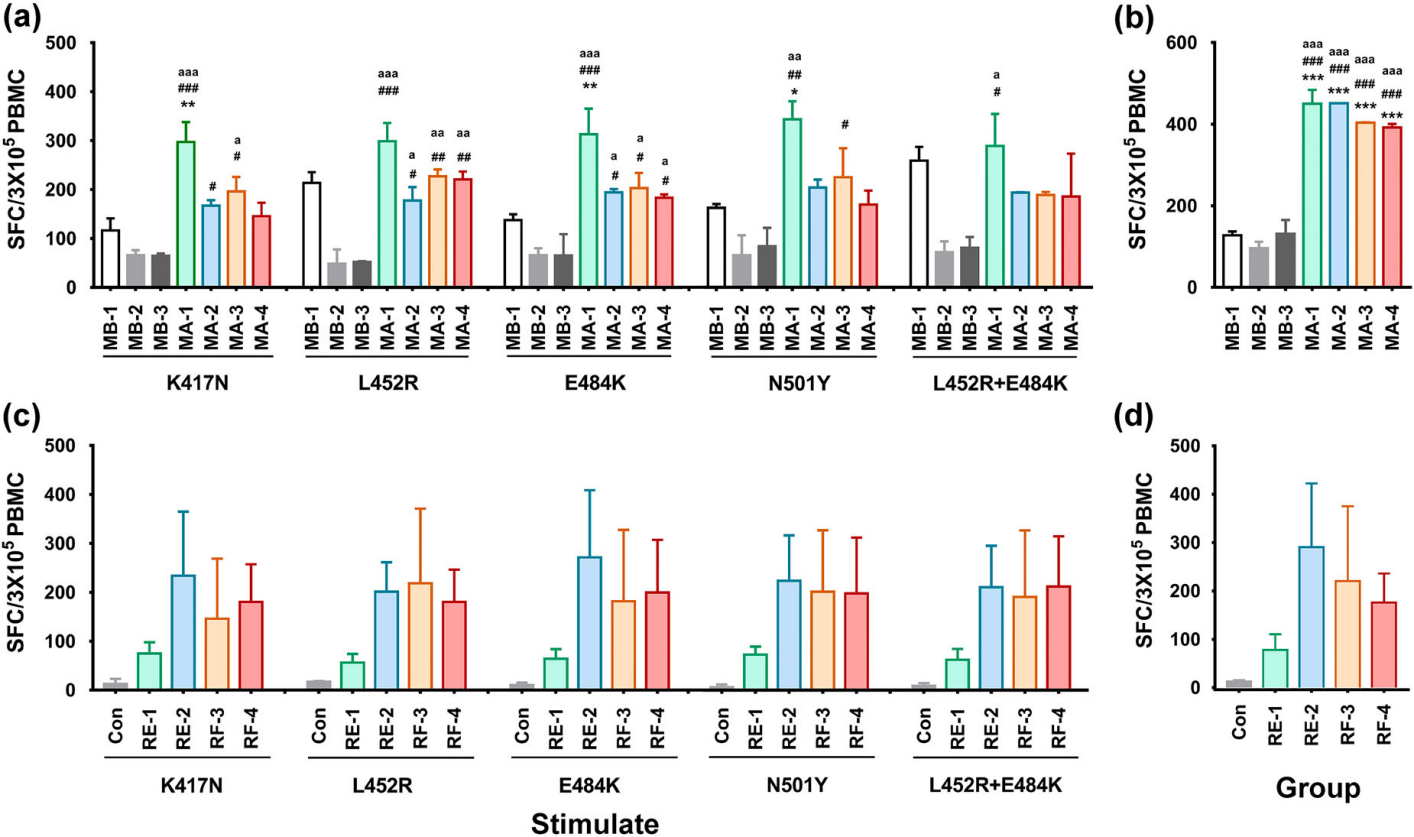
Development of a specific T cell response against different antigens from variants in individuals immunized with the heterologous vaccine administered via the ID route. (a) Specific T cell responses against RBD protein with five variants (four single-point mutation RBD proteins and a single two-point mutation RBD protein) were induced in immunized mice of the MA group compared to the MB group. (b) Specific T cell responses against N proteins were induced in immunized mice of the MA groups compared to MB groups. (c) Specific T cell responses against RBD proteins with five variants (four single-point mutation RBD proteins and a single two-point mutation RBD protein) were induced in immunized macaques from the RE and RF groups. (d) Specific T cell responses against N proteins were induced in immunized macaques in the RE and RF groups. The blood samples were obtained on day 28 after boosting with the K-S antigen. In the monkey experiment, blood samples were obtained from three untreated, healthy macaques included in the control group (Con). Statistical significance was assessed by two-way ANOVA. *, p < 0.05; **, p < 0.01;***; p < 0.001 versus MB-1 group; #, p < 0.05; ##, p < 0.01; ###, p < 0.001 versus MB-2 group; a, p < 0.05; aa, p < 0.01; aaa, p < 0.001 versus MB-3 group in WT mice (n = 12 per subgroup). There was no significant difference in monkeys between different subgroups (n = 4 per subgroup and n = 3 in control).

In our previous study on SARS-CoV-2 inactivated vaccines, memory T cell responses against the S and N antigens of the Wuhan strain were identified in vaccinated animals and humans [19,29]. Herein, we aimed to investigate whether the immune memory induced by intradermal immunization with the inactivated vaccine could be enhanced for distinct recognition of variant antigens after boosting with the K-S antigen with mutated sites. An IFN-γ ELISPOT assay with variant antigens, including RBD proteins with the 417N, 452R, 484Q, 501Y, and 452R+484Q mutations, was used in the current study, which showed that specific T cell responses against RBD proteins from five variants were significantly stronger in mice immunized with the inactivated vaccine in group MA on day 28 after booster with the K-S antigen than mice in the adjuvant control (MB-2) and K-S antigen alone control groups (MB-1) (Figure 5a); there were no significant differences between groups based on antigen quantity and number of immunizations (Figure 5a). The T cell response against the N protein in group MA also showed a strong upward trend (Figure 5b). The ELISPOT results for macaques suggested a dynamic upward trend of T cell responses against the five variants compared to the control on day 28 after booster with the K-S antigen, which showed a dose-effect relationship in the RE group (Figure 5c). The T cell response against the N protein in these macaques also presented a similar trend (Figure 5d). Together with the antibody response data, these results suggest that the heterologous strategy using the vaccine was suitable for the prevention of infection caused by variants through the induction of systemic immunity with a high titer of neutralizing antibodies and specific T cell recognition.

### Evaluation of the immunological protective efficacy of the heterologous vaccine strategy in hACE2 transgenic mice after viral challenge

**Figure 6.**
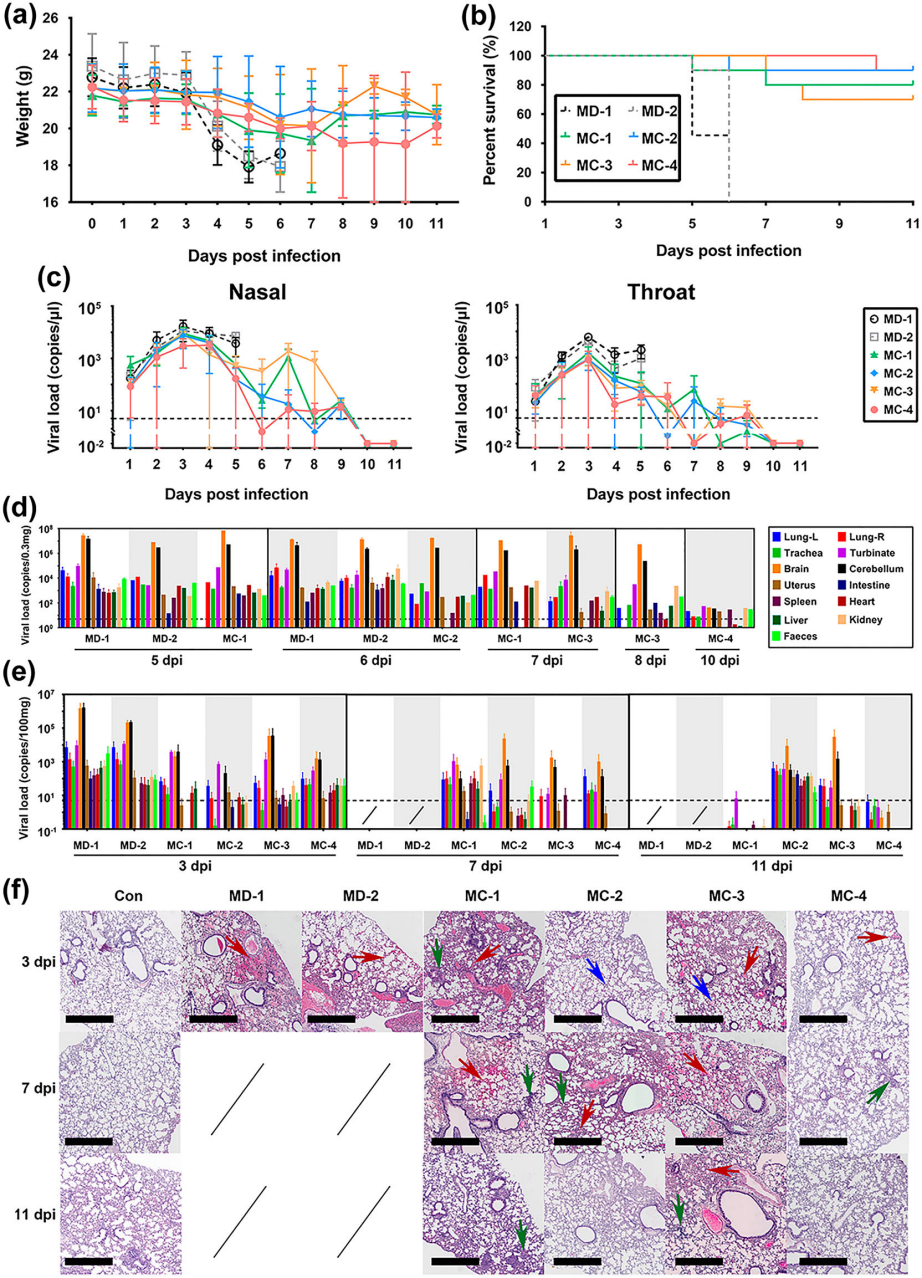
Immunity elicited in hACE2 transgenic mice by the heterologous vaccine administered intradermally enables the prevention of infection caused by the B.1.617.2 strain. (a) Body weight changes during viral challenge. In the MC and MD groups, hACE2 transgenic mice were immunized with the heterologous vaccine administered intradermally (n = 10 per subgroup as observation set). (b) Percent survival during viral challenge in the MC and MD groups (n = 10 per subgroup as observation set). (c) Viral loads of the various tissues or organs from the dead mice during viral challenge in the MC and MD groups. The detection threshold (5 copies/μl) is shown by the dotted line (n = 12 per subgroup). (d) Viral loads in the nasal and oral cavities during viral challenge in the MC and MD groups. The detection threshold (5 copies/μl) is shown by the dotted line. Because all mice in the MD-1 and MD-2 groups died by day 6 after infection, the viral load data were not obtained (n = 12 per subgroup). (e) Viral loads in various organs and tissues from euthanized mice as planned during the viral challenge in the MC and MD groups. The detection threshold (5 copies/μl) is shown by the dotted line. Samples of organs and tissues were obtained on days 3, 7, and 11 after viral infection. Because all mice in the MD-1 and MD-2 groups died by day 6 after infection, data on viral load are not available (n = 12 per subgroup). (f) Pathological observation of the lungs during viral challenge in the MC and MD groups. Samples of organs and tissues were obtained on days 3, 7, and 11 after viral infection. Bleeding (red arrow), edema (blue arrow), and alveolar wall thickening (green arrow) are shown. Bar, 600 μm (n = 12 per subgroup). Statistical significance was assessed by two-way ANOVA. Data are expressed as mean ± SD.

Although our data on antibody and T cell responses are robust, the clinical protective efficacy of this heterologous vaccine required further evaluation. Therefore, a viral challenge test with the B.1.617.2 strain was performed in immunized hACE2 transgenic mice from groups MC and MD according to the schedule shown in Figure 1a. Virus (10^3^ CCID_50_/each) was inoculated by a nasal spray. Clinical observation of the challenged mice within 11 days after the infection suggested that the adjuvant (MD-2) and K-S antigen (MD-1) control mice and a few MC-1 and MC-3 mice developed disease manifestations, such as arched back, shedding of hair, and body weight loss, to varying degrees (Figure 6a). In addition, some mice died between days 5 and 8. In contrast, the MC-2 mice received a single intradermal injection of 50 U of inactivated vaccine followed by a booster with the K-S antigen. These mice were healthy, except for one mouse that died. The mice in MC-4 received two intradermal injections of 50 U of inactivated vaccine followed by a booster with the K-S antigen. These mice were healthy during the observation period, except for one mouse that died when bitten by other mice (Figure 6b). The death of these mice suggested high viral proliferation in the lung and brain tissues of animals, especially in the MD-1 and MD-2 groups (Figure 6d). Further monitoring of the viral loads in surviving mice indicated that the mice in subgroups MC-2 and MC-4 had only 1.73–169.29 copies/μl and 0.38–49.64 copies/μl in the nose and throat, respectively, on days 5 and 6. Most mice had viral loads close to the detection threshold (5 copies/μl) compared to the obvious upward trend in the other subgroups, which reached 25.58–526.36 copies/μl and 11.52–103.71 copies/μl in the MC-3 and MC-1 subgroups, respectively (Figure 6c). Further detection of viral loads in various organs and tissues of the sacrificed mice on days 3, 7, and 11 confirmed that the mice in MC-2 and MC-4 were protected by the immunity induced by the heterologous vaccine (two intradermal injections of 50 U of inactivated vaccine followed by a booster with the K-S antigen) in the challenge test with B.1.617.2 strain (Figure 6e). The histopathological analysis confirmed these results by comparing the pathological injuries in various organs and tissues, especially the lung tissues, between MC-2 or MC-4 and the other subgroups (Table 1; Figure 6f). These data strongly suggest that both strategies of intradermal immunization (i.e. “1 + 1” and “2 + 1”) with the inactivated vaccine and K-S antigen led to effective immunity against SARS-CoV-2 variants, high levels of neutralizing antibodies, and developing specific T cell recognition ability.

**Table 1.** Histopathological analysis of various tissues or organs in hACE2 transgenic mice immunized with the heterologous vaccine administered intradermally after B.1.617.2 strain challenge.

Organ	Group	Day post infection		
		3	7	11
Brain	MB-1	−	/	/
	MB-2	−	/	/
	MA-1	−	−∼+	−∼+
	MA-2	−	−∼±	−
	MA-3	−	−	−∼+
	MA-4	−	−	−
Heart	MB-1	−	/	/
	MB-2	−∼++	/	/
	MA-1	−	−	−
	MA-2	−∼±	−	−
	MA-3	−	−	−
	MA-4	−	−	−
Liver	MB-1	+	/	/
	MB-2	+	/	/
	MA-1	−∼±	−∼±	−∼±
	MA-2	±∼+	±∼+	±∼+
	MA-3	−∼+	−∼±	±∼+
	MA-4	−∼±	−∼+	−∼±
Spleen	MB-1	−	/	/
	MB-2	−	/	/
	MA-1	−	−	−
	MA-2	−	−	−
	MA-3	−	−	−
	MA-4	−	−	−
Kidney	MB-1	−	/	/
	MB-2	−	/	/
	MA-1	−	−	−
	MA-2	−	−	−
	MA-3	−∼±	−	−
	MA-4	−	−	−
Gnitals	MB-1	−	/	/
	MB-2	−	/	/
	MA-1	−	−	−
	MA-2	−	−	−
	MA-3	−	−	−
	MA-4	−	−	−

Note: −, no significant pathological changes; ±, very light pathological changes; +, light pathological changes; ++, mild pathological changes; +++, severe pathological changes

## Discussion

The recently emerged SARS-CoV-2 variants have led to concern from the public and researchers. The global pandemic of COVID-19 has been partially controlled in most countries of the world [2–5]. Due to the new SARS-CoV-2 variants, rapidly declining neutralizing antibodies in convalescent patients [9,10], concerns about the persistence of neutralizing antibodies in vaccinated individuals, and a lack of understanding of the immunological characteristics of this virus, it is important to investigate new generations of vaccines effective against these variants based on the licensed vaccines administered to more than 1 billion people worldwide [1,30]. Herein, we proposed a strategy of intradermal immunization with a heterologous vaccine composed of 1 or 2 doses of inactivated vaccine and a booster with the S1 protein with mutations (K-S), which was designed to increase the immune efficacy of licensed vaccines by increasing the neutralizing antibody titers and promoting specific T cell responses against variant antigens. First, our study suggested that only 1/5–1/3 of the antigen quantity previously used in the inactivated vaccine followed by a booster with the K-S antigen (10 µg/dose) in mice and macaques was capable of augmenting the neutralizing antibody titers to 5–10 times those observed in individuals vaccinated with two intramuscular injections of the inactivated vaccine. In mice, this upward trend of antibody titers showed a positive relationship with the quantity of antigen and number of immunizations with the inactivated vaccine. In macaques, this relationship was stronger than that in mice. These data support the conclusion that the intradermal K-S booster enhanced the induction of an immune response in both animals. Second, mice and macaques immunized with this heterologous vaccine showed stronger specific T cell responses against 5 variants of RBD proteins, with similar reactivity levels associated with stronger responsive spots against the N antigen. These data suggest that a higher titer of neutralizing antibody primarily depends on systemic immunity, as evidenced by the sensitive and specific antigenic recognition ability of memory T cells against S and N antigen variants. Similarly, previous studies in which heterologous vaccines, including DNA vaccines combined with S antigen boosters [31] and inactivated vaccines followed by protein boosters, also suggested that this strategy induces higher antibody and CTL responses in animals, including humans [32,33]. Third, the viral challenge test with the B.1.617.2 strain confirmed that both schedules (i.e. “1 + 1” and “2 + 1”) produced a protective effect against this strain. However, the data showed significantly low viral load in tissues of immunized mice and little or slight inflammatory infiltration in lung and brain tissues compared to those in the controls. However, the antigen content of the inactivated vaccine is a more powerful determinant of the immunological protective effect than the number of immunizations. These results suggest that our strategy of using heterologous vaccines against new variants was feasible, enhanced the anamnestic immune response, and elicited specific recognition of variants by T cells in the immunized population, especially those vaccinated with inactivated vaccines. This heterologous prime-boost strategy has been evaluated for its effectiveness and safety in this work and previous studies and has been confirmed to be effective in previous studies [34–36]. Importantly, the present study provided important evidence for intradermal immunization, which was recommended for the inactivated polio vaccine by the WHO because of the reduced quantity of antigen used in children globally [37]. Our work suggests that the strategy of intradermal immunization can not only increase the supply of vaccines to meet the global demand for controlling the COVID-19 pandemic but also imply a possible mechanism by which a booster with the S1 protein enables the induction of an immune response to inactivated vaccines via effective recall of immune memory. It should be mentioned that this approach will be useful for large populations vaccinated with inactivated vaccines to improve their immunity against SARS-CoV-2 variants by increasing the levels of neutralizing antibodies and promoting specific recognition of antigens from variants. Further studies on the genetic prediction of variation trends in the S protein of SARS-CoV-2 and effective vaccine strategies for antigen boost, such as by using proteins or mRNAs, will be of interest.

In conclusion, this study reports a new heterologous prime-boost strategy of intradermal immunization with the inactivated vaccine and K-S antigen. This strategy can significantly increase the neutralizing antibody titers and promote specific T cell responses in mice and macaques. Importantly, this strategy is efficient in eliciting immune protective effects against viral variants. Heterologous immunization via the intradermal route may be useful to protect against the rapid emergence of SARS-CoV-2 variant, even though the underlying mechanism requires further investigation.

## Supplementary Material

Supplemental Material
