# Supplementary material for: Preclinical immunological evaluation of an intradermal heterologous vaccine against SARS-CoV-2 variants
Source: Emerg Microbes Infect. 2022 Jan 7;11(1):212–26. doi: 10.1080/22221751.2021.2021807 (PMC8745378; doi:10.1080/22221751.2021.2021807)
Supplement: Supplemental Material [file TEMI_A_2021807_SM9876.docx]

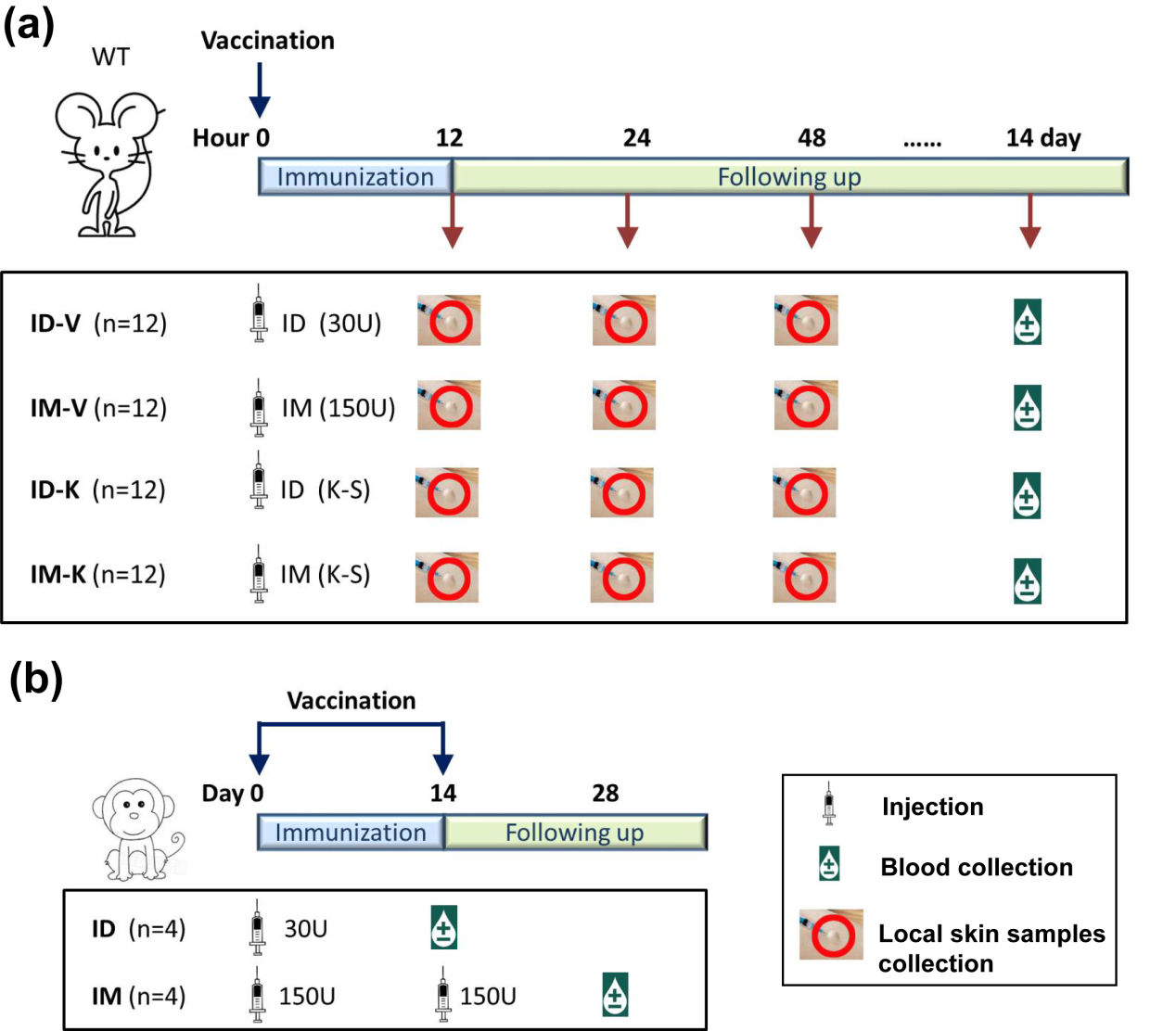


**Figure S1. Comparison of the effects of animal ID and IM immunization schedule.**

1. Mouse immunity experiment. The WT mice received single ID or IM immunization (syringe signs) with inactivated SARS-CoV-2 vaccine (30 U for ID and 150 U for IM) and K-S antigen (10 μg/dose). Local skin samples (red circle signs) were obtained at 12, 24, and 48 h after the first immunization. Blood samples (water drop-like signs) were obtained on day 14 after the first immunization for antibody assays.
2. Rhesus macaque immunity experiment. Macaques received single ID immunization (syringe signs) with 30 U of inactivated vaccine or two IM immunizations with 150 U of inactivated vaccine at an interval of 14 days. Blood samples (water drop-like signs) were obtained on day 14 after the final immunization for antibody assays.


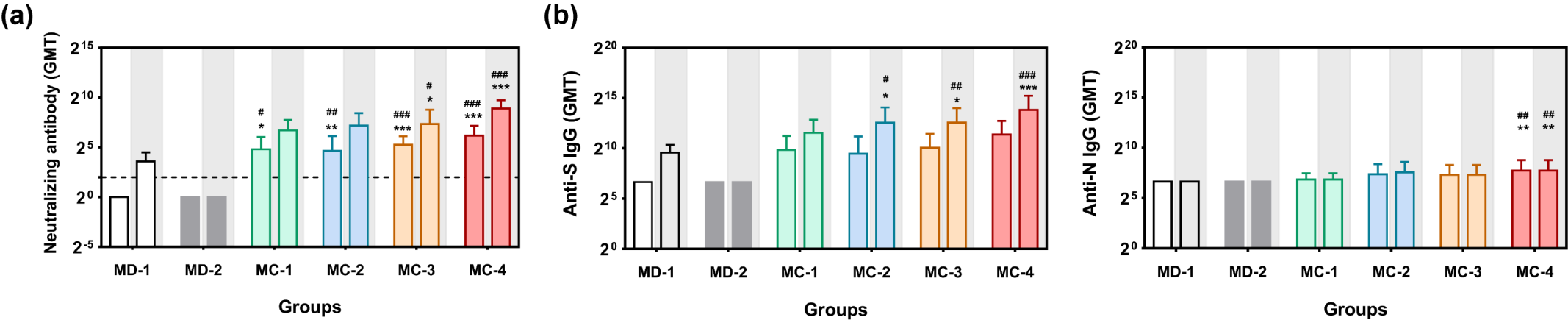


**Figure S2. The immune responses induced by intradermal immunization with inactivated vaccine and K-S protein in hACE2 transgenic mice.**

1. Neutralizing antibodies recalled by boosting with the K-S protein in hACE2 transgenic mice immunized with the inactivated vaccine. The live viral strain is KMS-2 (Wuhan strain).
2. The levels of anti-S and anti-N antibodies after booster dose with the K-S protein in hACE2 transgenic mice immunized with the inactivated vaccine.

Data in the column chart with white color at the bottom are the antibody titers before booster dose with K-S protein (at day 28 after the first immunization) and those with gray color at the bottom are the antibody titers 14 days after the booster immunization (at day 42 after the first immunization). Statistical significance was assessed by two-way ANOVA. *, p < 0.05; **, p < 0.01;***; p < 0.001 versus MD-1 group. #, p < 0.05; ##, p < 0.01; ###, p < 0.001 versus MD-2 group.

**Table S1. The primers used in this study.**

| **Primer** | **Sequence (5'to3')** |
| --- | --- |
| 4-1BBL-F | AAGCCTCAGGTAGATGACT |
| 4-1BBL-R | GAACGGTCCACTAACTTGT |
| BTLA-F | GCCAGGACAGGAGAGTTA |
| BTLA-R | CTTACACCAAGTCACATTAGG |
| CD160-F | CCTGAGACCAACTTAGAACA |
| CD160-R | AACACCAACTGAGATGACTT |
| GITRL-F | CCAAGGTGTCCAGAATGAA |
| GITRL-R | TGAGATGAACTCTTCCAACA |
| IKK-α-F | TGGAAGAGACTGCTGACA |
| IKK-α-R | ATGAAGAACACTTGCTGAGA |
| IKK-β-F | CTCCGAAGATACTTGAACCA |
| IKK-β-R | CGATGCGATGTCACTCAG |
| LIGHT-F | TTCTGAGCACCTACATTCC |
| LIGHT-R | CTTCTGACCAACCATTCCT |
| LTα3-F | ACTGCCGCTTCCTCTATA |
| LTα3-R | GTGTCATGTGGAGAACCT |
| RANKL-F | TCACTCTGTCCTCTTGGTA |
| RANKL-R | CAGGTAATAGAAGCCATCTTG |
| TAK-F | CCATCACTTACACAGCAATC |
| TAK-R | CCTCACAGATACATACACAGA |
| TNFα-F | GTGGAACTGGCAGAAGAG |
| TNFα-R | GGTGAAGGTCGGTGTGAACG |
| IFN-α-F | CTTCCTCAGACTCATAACCT |
| IFN-α-R | AGTCCTTCCTGTCCTTCA |
| IFN-β-F | AACTCCACCAGCAGACAG |
| IFN-β-R | GAGAGCAGTTGAGGACATC |
| GAPDH-F | GGTGAAGGTCGGTGTGAACG |
| GAPDH-R | CTCGCTCCTGGAAGATGGTG |
| TLIA-F | AATAAGCAACAACTGGTTCC |
| TLIA-R | ATTAGTCTGTCTCCTTCTTCC |
| M IL-5 F | CTCTGTTGACAAGCAATGAGACG |
| M IL-5 R | TCTTCAGTATGTCTAGCCCCTG |
| M IL-13 F | CCTGGCTCTTGCTTGCCTT |
| M IL-13 R | GGTCTTGTGTGATGTTGCTCA |
| M IL-9 F | ATGTTGGTGACATACATCCTTGC |
| M IL-9 R | TGACGGTGGATCATCCTTCAG |
| M IL-2 F | TGAGCAGGATGGAGAATTACAGG |
| M IL-2 R | GTCCAAGTTCATCTTCTAGGCAC |
| M IL-4 F | GGTCTCAACCCCCAGCTAGT |
| M IL-4 R | GCCGATGATCTCTCTCAAGTGAT |
| M IL-7 F | TTCCTCCACTGATCCTTGTTCT |
| M IL-7 R | AGCAGCTTCCTTTGTATCATCAC |
| M IL-25 F | ACAGGGACTTGAATCGGGTC |
| M IL-25 R | TGGTAAAGTGGGACGGAGTTG |
| M IL-33 F | TCCAACTCCAAGATTTCCCCG |
| M IL-33 R | CATGCAGTAGACATGGCAGAA |
